# Mitochondria-associated gene expression perturbation predicts clinical outcomes and shows potential for targeted therapy in neuroblastoma

**DOI:** 10.3389/fped.2023.1094926

**Published:** 2023-03-21

**Authors:** Chengwei Chai, Yan Chen, Yuanyuan Luo, Hong Zhang, Zhihua Ye, Xiaobing He, Yan Zou, Yingyi Xu, Le Li, Jue Tang, Qiang Wu

**Affiliations:** ^1^ Department of Pediatric Surgery, Guangzhou Women and Children's Medical Center, Guangzhou Medical University, Guangzhou, China; ^2^ Guangdong Provincial Key Laboratory of Research in Structural Birth Defect Disease, Guangzhou Women and Children's Medical Center, Guangzhou Medical University, Guangzhou, China; ^3^ Department of Anesthesiology, Guangzhou Women and Children’s Medical Center, Guangzhou Medical University, Guangzhou, China

**Keywords:** neuroblastoma, mitochondria associated protein, targeted therapy, clinical outcome, multi-omic

## Abstract

**Background:**

Mitochondria have long been considered a potential target in cancer therapy because malignant cells are known for their altered energy production. However, there is a lack of comprehensive research on the involvement of mitochondria-associated proteins (MAPs) in neuroblastoma (NB), and their potential as therapeutic targets is yet to be fully explored.

**Methods:**

MAP genes were defined based on the protein-coding genes with mitochondrial localization. The mRNA expression patterns and dynamics of MAP genes associated with NB were investigated by integrating publicly available transcriptional profiles at the cellular and tissue levels. Multivariate Cox regression analysis was conducted to reveal the association of MAP genes with the overall survival (OS) and clinical subgroups of NB patients. The single-cell RNA-seq dataset and gene dependency screening datasets were analyzed to reveal the therapeutic potential of targeting MAP genes.

**Results:**

We compiled a total of 1,712 MAP genes. We found the global and cell type-specific mRNA expression changes of the MAP genes associated with NB status and survival. Our analyses revealed a group of MAP gene signatures independent of *MYCN*-amplification status associated with NB outcome. We provided computational evidence with selected MAP genes showing good performance in predicting long-term prognosis. By analyzing gene dependency of the MAP genes in NB cell lines and *ex vivo* human primary T cells, we demonstrated the therapeutic potential of targeting several MAP genes in NB tumors.

**Conclusions:**

Collectively, our study provides evidence for the MAP genes as extended candidates in NB tumor stratification and staging, prognostic prediction, and targeted drug development.

## Background

Childhood neuroblastoma (NB), one of the most common extracranial solid tumors in children, accounts for approximately 6%∼10% of all childhood cancers (1). Although NB has been revealed to arise from the precursor cells of the sympathetic nervous system and adrenal medulla (2, 3), the clinical course of NB is highly heterogeneous, which poses a challenge for NB therapy, particularly for high-risk patients whose long-term survival is below 50%. Advances in our understanding of the relevant clinical and biological features have made it possible to more accurately stratify tumor risk and improve NB treatment (4). A unified clinical consensus on NB treatment recommends combining multiple molecular markers (5).

The understanding of NB development, risk classification, and tumor staging have been improved through many tumor genetic analyses, which revealed the importance of segmental chromosomal alterations (SCAs) and specific genetic variants. Different SCAs have been found, including loss of 1p (6), 3p (7), 4p (6), 6q (8), and 11q (9), and gain of 1q (10), 2p (11), and 17q (12). The specific genetic variants associated with the outcome of NB patients include amplification of *MYCN* (13), *DDX1* (14), *NAG* (15), and *ALX* (16), and mutations in genes *CASC15, BARD1, CHEK2, LMO1, LIN28B, AXIN2, BRCA1, TP53, SMARCA4*, and *CDK1NB* (17). Despite such genetic findings, only a few have been applied in clinical practice. *MYCN* amplification is one of the most studied in predicting the prognosis of NB patients. High-throughput technologies advanced translational research in clinical oncology by allowing us to explore the NB at different molecular levels. Gene expression signatures derived from the transcriptomes associated with NB subgroups can better characterize tumors' molecular profile and heterogeneity. In addition, integrating different gene expression datasets can result in better NB patient stratification compared to using these datasets individually (18).

Cancer cells are partially characterized by reprogrammed energy generation; Therefore, studying mitochondrial-related gene expression and regulation is of particular significance for revealing NB prognosis and developing a potential target in cancer therapy. However, a comprehensive assessment of the role of mitochondria-associated proteins (MAPs) in NB has yet to be conducted. Here, by integrating publicly available high-throughput transcriptional profiles at the bulk tissue-level and single cell-level resolutions, we systematically analyzed the MAP gene expression dynamics in NB. We found the global and cell-type-specific mRNA expression changes of the MAP genes associated with NB status and OS. Our analyses revealed MAP gene signatures independent of *MYCN*-amplification status associated with the clinical outcome of NB patients. We provided computational evidence for selected MAP genes showing good performance in predicting long-term prognosis. By analyzing gene dependency or essentiality for cell proliferation and survival of these MAP genes in NB cell lines and human primary T cells, we demonstrated the therapeutic potential of targeting several MAP genes in NB tumors. Collectively, our study shows that MAP genes could be potential candidates for staging tumors, predicting the prognosis of NB, and developing targeted drugs for the disease.

## Methods

### Collection and processing of datasets

**Dataset 1**: cell line-based bulk RNA-seq data, obtained from the Gene Expression Omnibus (GEO) database (19) under accession number GSE89413 (20). This dataset is comprised of 39 commonly used NB cell lines and the other two control samples: the hTERT-immortalized retinal pigmented epithelial cell RPE-1, which is widely used as a non-neuroblastoma control, and the cells from the pooled fetal brain, which can serve as a non-neuroblastoma neural-cell derived control.

Fragments Per Kilobase of transcript per Million mapped reads (FPKM) values were downloaded and converted to TPM, short for transcripts-per-million, following the formula: TPM*_i_* = FPKM*_i_*/Sum(FPKM*_j_*)*10^6^, where *i* denotes the *i*-th gene and *j* denotes the *j*-th subject. We further excluded low-expressed genes with the averaged TPM ≤ 3 across all the samples. Log2-transformed (TPM + 1) values were used and further normalized with the *normalize.quantile* function in the “preprocessCore” (v1.50.0) package. Differential expression analysis based on the linear model by weighted least squares was conducted with the “limma” package (25), and genes with the adjusted *P* values < 0.05 were identified to be differentially expressed.

**Dataset 2**: primary NB tissue-based bulk RNA-seq data from the RNA Sequencing Quality Control (SEQC) cohort, downloaded from the GEO under accession number GSE49711 and GSE62564 (21). This dataset contained gene expression profiles of 498 NB patients and the corresponding clinical information, including *MYCN* status, clinical risk level (high or low), disease stage according to International Neuroblastoma Staging System (INSS) (1, 2, 3, 4, and 4S) (22), the occurrence of a tumor progression event (yes = 1; no = 0), and occurrence of death from the disease (yes = 1; no = 0).

In this project, gene expression was quantified using log base 2 of the number of bases aligned in the gene, divided by the number of terabases aligned in known genes and by the length of the gene (21). We directly downloaded this gene expression matrix. 1,259 MAP genes were included. Principle component analyses (PCA) of the MAP gene expression matrix subset were conducted and visualized with the functions from the “FactoMineR” (v2.4) and “factoextra” (v.1.0.7) packages. Differential expression analyses were conducted between subgroups for each clinical parameter with the “limma” package.

**Dataset 3**: single-cell RNA-seq (scRNA-seq) dataset of NB tissues, downloaded from the GEO under accession number GSE137804 (2). Raw counts of single cells were obtained. We first excluded low-quality cells with less than 500 genes detected and more than 10% of genes derived from the mitochondrial genome. The filtered gene expression matrix was then normalized with Seurat's *NormalizeData* function (23), in which feature counts for each cell were divided by the total counts for that cell, multiplied by the scale factor (10,000), and then subjected to natural-log transformation using log1p. Highly variable genes were identified and used for the subsequent PCA, which was performed using graph-based clustering and visualized using *t*-Distributed Stochastic Neighbor Embedding (*t*-SNE) or Uniform Manifold Approximation and Projection (UMAP) with the *RunTSNE* and *RunUMAP* functions. We integrated the cells from each NB tumor with Seurat's *FindIntegrationAnchors* and *IntegrateData* functions.

**Dataset 4**: MAP gene dependency of cell viability dataset, downloaded from the Dependency Map (DepMap) portal (v21Q3, https://depmap.org/portal/) (24), one of the largest collections of CRISPR screening studies in 1,054 kinds of human cancer cells from 30 lineages. Genome-wide gene dependency scores were quantified using CERES method by adjusting copy number amplification effect, where the scores of 0 and −1 represent the median effects of nonessential genes and common core essential genes, respectively (25). In this study, we used a cutoff of −0.5 to define cellular dependency and essentiality of the MAP genes in 31 cell lines belonging to the NB lineage.

**Dataset 5**: Genome-wide CRISPR screens in primary human T cells for identifying gene targets that regulate T cell proliferation in response to T cell receptor stimulation. The sgRNAs targeting specific genes were defined to be regulators of T cell proliferation by calculating the abundance-based rank difference between the highly dividing cells and non-dividing cells (26).

### MAP gene collection

The MAP genes were extracted and combined from the MitoCarta (v3.0; https://www.broadinstitute.org/mitocarta) (27) and the human protein atlas (HPA; https://www.proteinatlas.org/) databases (28). Specifically, the MitoCarta3.0 is an inventory of 1,136 human and 1,140 mouse genes encoding proteins with support of mitochondrial localization, now with sub-mitochondrial compartment and pathway annotations; the cell atlas of the HPA project contains 1,156 genes which have been shown to localize to mitochondria.

### Mapping MAP genes to MitoPathways

According to the MitoCarta database, MAP genes were annotated to eight major Mitochondrial Pathways (MitoPathways), including mitochondrial central dogma, protein import and sorting, protein homeostasis, oxidative phosphorylation (OXPHOS), metabolism, small molecule transport, signaling, and mitochondrial dynamics and surveillance (29). For the MAP genes exclusively in the HPA database, we conducted Gene Ontology (GO)-based gene-level semantic similarity analysis with the GOSemSim package (v2.14.2) (30). Each of these genes was annotated to the MitoPathway with the highest similarity score with the *clusterSim* function by comparing the target genes to those annotated in each MitoPathway with parameters: *measure="Wang”, combine=” BMA”*. The genes annotated to >3 sub-pathways were excluded from our annotation pipeline. We obtained a total of 1,191 MAP genes annotated to the eight MitoPathways.

### Collection of putative target genes of MYC transcription factor

This gene set was downloaded from the Gene Set Enrichment Analysis website (https://www.gsea-msigdb.org/) (31) with the standard gene set name of DANG_MYC_TARGETS_UP, which was defined by the genes upregulated by MYC and whose promoters are bound by MYC, according to MYC target Gene Database. This gene set contains 144 members mapped to 130 genes.

### Pan-cancer analysis of MAP genes

The mRNA expression data sets generated by The Cancer Genome Atlas (TCGA) project (32) were downloaded using the UCSCXenaTools package (33). Sample barcode was annotated with the TCGAutils package (34). We used the Level-3 gene expression data sets from 21 cancer types, including the LUAD (Lung Adenocarcinoma), ACC (Adrenocortical Cancer), CHOL (Bile Duct Cancer), BLCA (Bladder Cancer), BRCA (Breast invasive carcinoma), CESC (Cervical Cancer), COAD (Colon adenocarcinoma), ESCA (Esophageal cancer), GBM (Glioblastoma), KIRC (Kidney Clear Cell Carcinoma), KIRP (Kidney Papillary Cell Carcinoma), LUSC (Lung Squamous Cell Carcinoma), SKCM (Melanoma), LIHC (Liver Cancer), LGG (Lower Grade Glioma), OV(Ovarian Cancer), UVM (Ocular melanomas), PAAD (Pancreatic Cancer), PRAD (Prostate Cancer), STAD (Stomach Cancer), and THCA (Thyroid Cancer). Because normal samples from the TCGA project are typically limited in the number for many cancer types, we used normal expression data from the GTEx project (https://www.gtexportal.org/). For GTEx datasets, we downloaded the “gene expression RNAseq—TOIL RSEM expected_count” matrix from the Xena project. GTEx samples were grouped according to the tissues corresponding to the TCGA cancers. Gene IDs and symbols were mapped and converted with the “org.Hs.eg.db” package (35). All the gene expression RNAseq datasets from Xena were generated with a same data processing pipeline to create a consistent meta-analysis of different datasets free of computational batch effects (36). Differential gene expression analyses were conducted with the “limma” package, and survival analyses were conducted with the GEPIA webserver (37).

### Functional enrichment analyses

We employed different methods for functional enrichment analysis based on the different gene lists we were concerned with as indicated in our manuscript. Gene-set enrichment analysis (GSEA) is a computational method that determines whether an *a priori*-defined set of genes shows statistically significant, concordant differences between two conditions. For dataset 1, we tested the enrichment of each MitoPathway gene set using the ranked MAP genes based on their mRNA fold changes values. Gene Ontology (GO) term analysis was performed by comparing the gene set of interest to a reference set of genes that have been annotated with GO terms. GSEA and GEO term analyses were performed with the *runGSA* and *runGSAhyper* functions, respectively, which were embedded in the piano package (38).

### Cox regression-based survival analyses and important gene/feature selection

A univariate Cox regression analysis was performed to reveal the association between the genes and the OS of NB with the “survival” package (v3.1.12) in the R environment. The genes with log-rank *P* < 0.05 were considered to be the prognosis-related genes and further subjected to a feature selection procedure. The mRNA expression levels of the tested genes in Cox regression analysis were separated into “high” and “low” groups based on the ranking of the genes across all the patients. A Lasso regression within a framework of five-fold cross-validation was conducted to reduce the variable dimension and select essential genes related to prognosis. The rated genes were further subjected to a multivariate Cox regression analysis. Cox regression and Lasso regression analyses were conducted with the “glmnet” package (v4.1–2) (39). The model assessment was performed using the time-dependent ROC analysis with the “timeROC” package (v0.4)(40).

## Results

### MAP mRNAs express differently in NB cell lines from normal cell types

**Figure 1:**
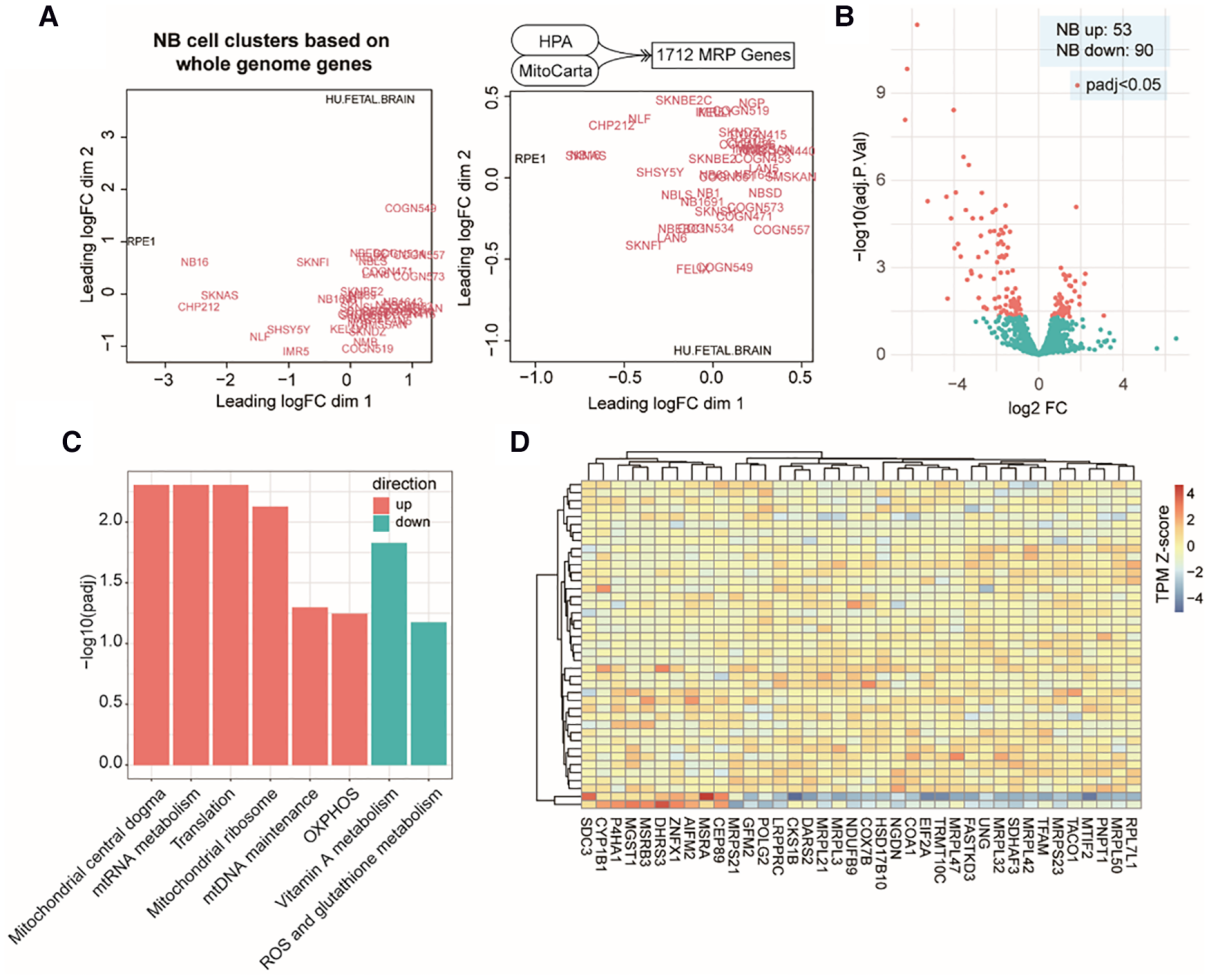
MAP mRNA expression changes in NB cell lines. (**A**). Multidimensional scaling plots of distances between gene expression profiles using the top 1,000 genes with the largest standard deviations between samples (left) or using all the MAP genes (right). Red colored labels indicate NB cell lines, black colored labels indicate the control samples. (**B**). Volcano plot of MAP gene expression changes in NB cell lines. (**C**). Bar plot of significant MitoPathways by GSEA analyses. Logarithmic adjusted *P* values are compared and plotted. Red bars indicate MitoPathways enriched by gene expression upregulation, and cyan bars indicate that enriched by gene expression downregulation. (**D**). Heatmap of normalized mRNA levels of highlighted MAP genes from significant pathways in NB cell lines and normal samples.

At the beginning of our study, we compiled a list of 1,712 MAP genes by integrating the HPA and the MitoCarta3.0 databases (see Methods). Using publicly available bulk RNA-Seq data containing 39 NB cell lines and two types of normal cells (Dataset 1, GSE89413; see Methods), clustering analyses based on the mRNA abundance of top variant genes and MAP genes showed a distinct separation between NB and normal cell types (Figure 1A), suggesting a different pattern of global gene expression in the NB cells and the ability of MAP genes to characterize NB cell identity. To further assess the gene expression alteration of these MAP genes in NB, we compared their mRNA levels between NB cells and normal ones and found 143 MAP genes (adjusted *P* < 0.05; Supplementary Table S1) showing significantly differential mRNA expression in NB cells (Figure 1B). Based on the MitoPathway annotation (see Methods), GSEA was performed to reveal the changes and preference of molecular pathways in NB cells. We found several gene sets that were significantly enriched (adjusted *P* < 0.1) by increased mRNA expression in NB cells, including the mitochondrial central dogma, mitochondrial RNA metabolism, translation, mitochondrial ribosome, mitochondrial DNA maintenance, and oxidative phosphorylation (OXPHOS). In contrary, the gene sets of vitamin A metabolism and ROS and glutathione metabolism were enriched by repressed mRNA expression in NB cells (Figure 1C). Of note, our differential expression analysis of MAP genes revealed some known mRNA expression biomarkers, such as *LDHB* (41), and *HSPD1* (42), whose upregulation was associated with NB tumor cell survival and prognosis. We also noted 38 genes annotated in the significantly changed pathways, including upregulation of mitochondrial DNA encoding genes (*MRPL50*, *MRPS23*, *MRPL42*, *MRPL32*, *MRPL47*, *MRPL3*, *MRPL21,* and *MRPS21*) and downregulation of *CEP89*, *AIFM2,* and *ZNFX1* in the NB cell lines (Figure 1D). These results indicated that mitochondria-related molecules undergo rewiring to create a supportive cellular environment for NB cells to survive, thus demonstrating the significance of monitoring the mRNA expression of MAP genes in NB cells.

### Characterizing MAP gene expression in primary NB tumors in different clinical subgroups

**Figure 2:**
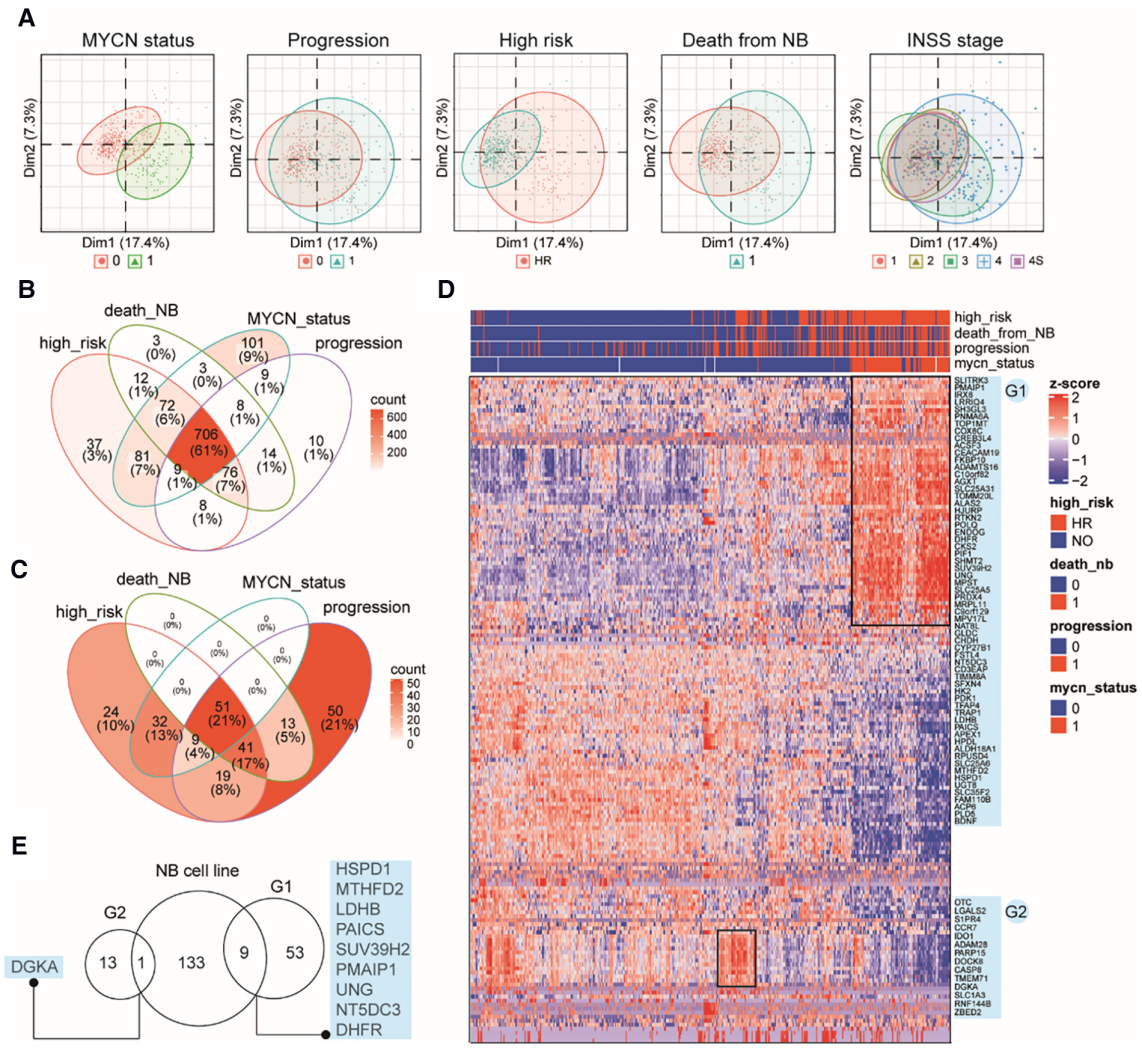
Characterizing MAP gene expression in primary NB tumors in different clinical subgroups. (**A**) Principal component analysis (PCA) plots of the top two PCs showing clustering relationship of 498 primary NB tumors by *MYCN* status, progression events, higher clinical risk, death from NB and INSS stages. (**B**) Venn diagram of differential MAP genes by comparing subgroups in four clinical parameters. (**C**) Venn diagram of NB tumors positive in four clinical parameters. (**D**) Heatmap of mRNA levels of top differential MAP genes (>2-fold) in NB tumors. Rows indicate MAP genes and columns indicate NB patients, both clustered by hierarchical clustering method. Clinical annotations for each patient are shown on the top of the heatmap. Two groups of MAP genes are highlighted and named as G1 and G2, shown on the right of the heatmap. (**E**) Venn diagram of MAP genes in G1, G2 and differential ones in the bulk cell line dataset.

Although immortalized cell lines represent the most widely used methods, they cannot reliably reflect the *in vivo* cellular environment of tumors because human tumor tissues contain a complex mixture of cell types and microenvironments (43). Therefore, we examined MAP gene expression dynamics in primary NB tumors using another publicly available bulk RNA-Seq data from 498 subjects (Dataset 2, GSE49711; see Methods). Interestingly, clustering analysis based on the MAP genes showed that NB tumors were grouped by *MYCN* status, progression events, high risk, or death from NB (Figure 2A), indicating that MAP gene expression were associated with amplified *MYCN*, tumor progression, higher clinical risk and higher risk of death of NB tumors. In contrast, we found a weak classification effect of the INSS stage information in NB tumors (Figure 2A), suggesting a weak correlation between global expression of MAP mRNAs and the four-level ordinal scale in the INSS stage, which was further excluded from our following analyses.

Differential gene expression analysis (DEA) was performed between subgroups for each of the remaining four parameters. A total of 1,149 MAP genes (Supplementary Table S2) were identified to be differentially expressed by combining the DEA results, where approximately 61% were shared by all four parameters, and 86% were shared by at least two parameters (Figure 2B), indicating that these clinical parameters have largely common molecular basis. This result was consistent with the clinical relevance between these parameters, as shown by overlapping the 239 NB tissues positive in high risk, progression occurrence, *MYCN* status, or death of the disease (Figure 2C). In order to thoroughly uncover these molecular changes, we employed hierarchical clustering analysis of NB tumors according to these four parameters and MAP genes with high fold changes (>2-fold). The analysis revealed NB subgroups and subgroup-specific MAP mRNA change signatures. Notably, our analysis highlighted two groups of MAP genes: G1 group (62 genes), which showed increased expression in concordance with *MYCN* amplification; and G2 group (14 genes), which was uniquely associated with NB tumors without *MYCN* amplification but with high clinical risk (Figure 2D). Because *MYCN* encodes the Myc oncogenic transcription factor, we compared the G1 genes to those putative Myc target genes that can be up-regulated by Myc and whose promoters are bound by Myc (see Methods), we found only three genes (*CKS2*, *APEX1*, *HSPD1*) shared between these two gene sets, indicating that the genetic regulatory networks underlying the genesis of NB are much more complicated than those related to the Myc regulatory network.

We noted that our analysis based on the primary NB tissues reproduced some known markers for NB prognosis and high-risk, including *CKS2* that was considered a prognostic marker of various tumors (44), *PDK1* that could provide significant hints for high-risk NB patients (45), and *PIF1*, one Myc-target gene, that could significantly repress tumor upon knockdown (46). We also compared the differential gene sets obtained from the bulk RNA-seq data of cell lines and primary tissues and found only ten common genes in these two groups, including *DGKA*, *HSPD1*, *MTHFD2*, *LDHB*, *PAICS*, *SUV39H2*, *UNG*, *NT5DC5*, and *DHFR* (Figure 2E), which indicated the importance of using tissue data to validate cell line-based data. Collectively, these results demonstrated that MAP genes also altered their expression pattern in primary NB tissues and showed a clinical potential to stratify NB patients.

### MAP genes exert cell type-specific mRNA expression in NB tumor microenvironment (TME)

**Figure 3:**
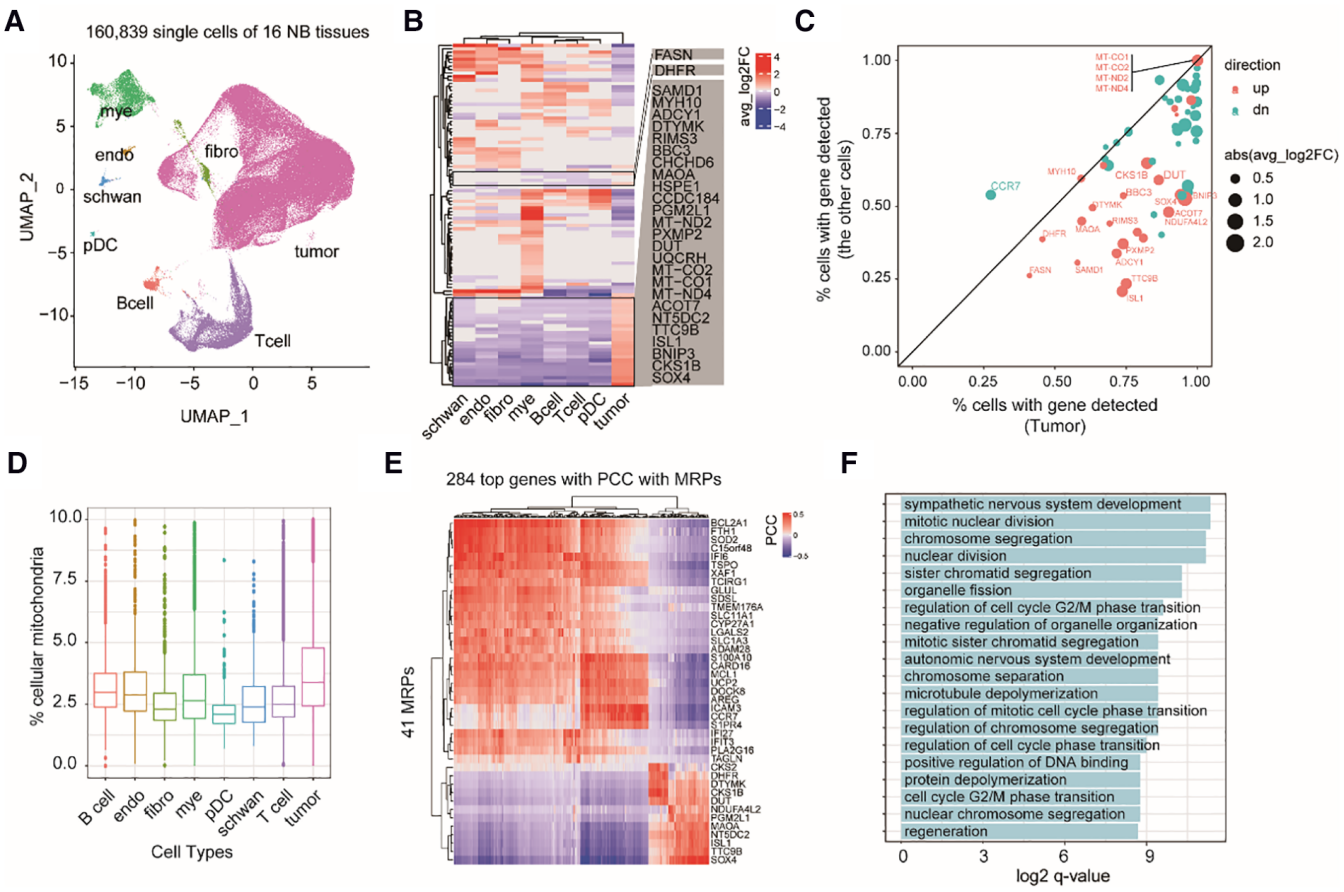
Exploring cell type-specific expression changes of MAP genes in NB TME. (**A**) UMAP projection of the single cell dataset colored according to cell type identity. Mye, myeloid cells; endo, endothelium cells; schwan, Schwann stromal cells; pDC, plasmacytoid dendritic cells; Bcell, B cells; Tcell, T cells; fibro, fibroblasts. (**B**) Heatmap of differential MAP genes in each cell type. (**C**) Scatter plot of the percentage of cells with detected expression of differential MAP genes in tumor and non-tumor cell types. Point size indicates the degree of gene expression foldchanges, point colors indicate up-regulation (red) and down-regulation (cyan) in the tumor cells. (**D**) boxplots of the percentage of shorted reads aligned to mtDNAs in each cell type. (**E**) Heatmap of 284 genes showing high correlation with MAP genes by pairwise gene expression correlation analysis across all the cells. (PCC > 0.4 or < −0.4). (**F**) Barplot of significant enriched GO terms by the top correlated genes with MAP genes.

Because tumor tissues are a complex mixture of cell types, we next asked whether the expression changes of these MAP genes are specific to certain cell types in NB TME or not. Therefore, we investigated 160,839 single-cell transcriptomes from 16 NB tissues (Dataset 3, GSE137804; see Methods). Cell identity was defined according to the original study (2), including eight major cell types: neuroendocrine cells (tumor cell), T cells, myeloid cells, B cells, Schwann stromal cells, fibroblasts, plasmacytoid dendritic cells (pDCs), and endothelium cells (Figure 3A). Comparing mRNA expression profiles between different cell types revealed 106 MAP genes showing diverged mRNA levels (adjusted *P* < 0.05; Supplementary Table S3). We identified 27 genes, which showed increasing mRNA levels specifically in tumor cells (Figures 3B,C). Comparing the percentage of cells with identified expression of these genes revealed that 22 out of 27 genes were mainly enriched in tumor cells (Figure 3C). We noted several mtDNA encoding genes, including *MT-CO1, MT-CO2, MT-ND2,* and *MT-ND4*, and nuclear DNA encoding genes but showing important roles in mitochondrial biogenesis, including *DUT* and *SOX4*. Accordingly, we inferred that mitochondrial production of NB tumor cells is accelerated. As expected, we observed a high abundance of short read counts mapping to mitochondrial DNA genes in tumor cells even after the removal of low-quality cells (Figure 3D; see Methods).

To uncover the relevant molecular functions involved in the complex ecosystem, we conducted gene co-expression analysis between the MAP and non-MAP genes across all of the single cells. We found 248 non-MAP genes showing a significant high correlation with MAP genes with Pearson correlation coefficient (PCC) > 0.4 or <−0.4 (Figure 3E). GO term analysis revealed that these correlated non-MAP genes were mainly from cell cycle-related cellular pathways (adjusted *P *< 0.05), such as mitotic nuclear division and chromosome segregation terms (Figure 3F). Taken together, our analyses suggested that MAP genes exert cell type-specific expression changes in NB tumors and possibly underlie the malignancy characteristics of tumor cells, such as an enhanced cell proliferation capacity than those non-tumor cells.

### MAP genes can predict the clinical outcome of NB patients

**Figure 4:**
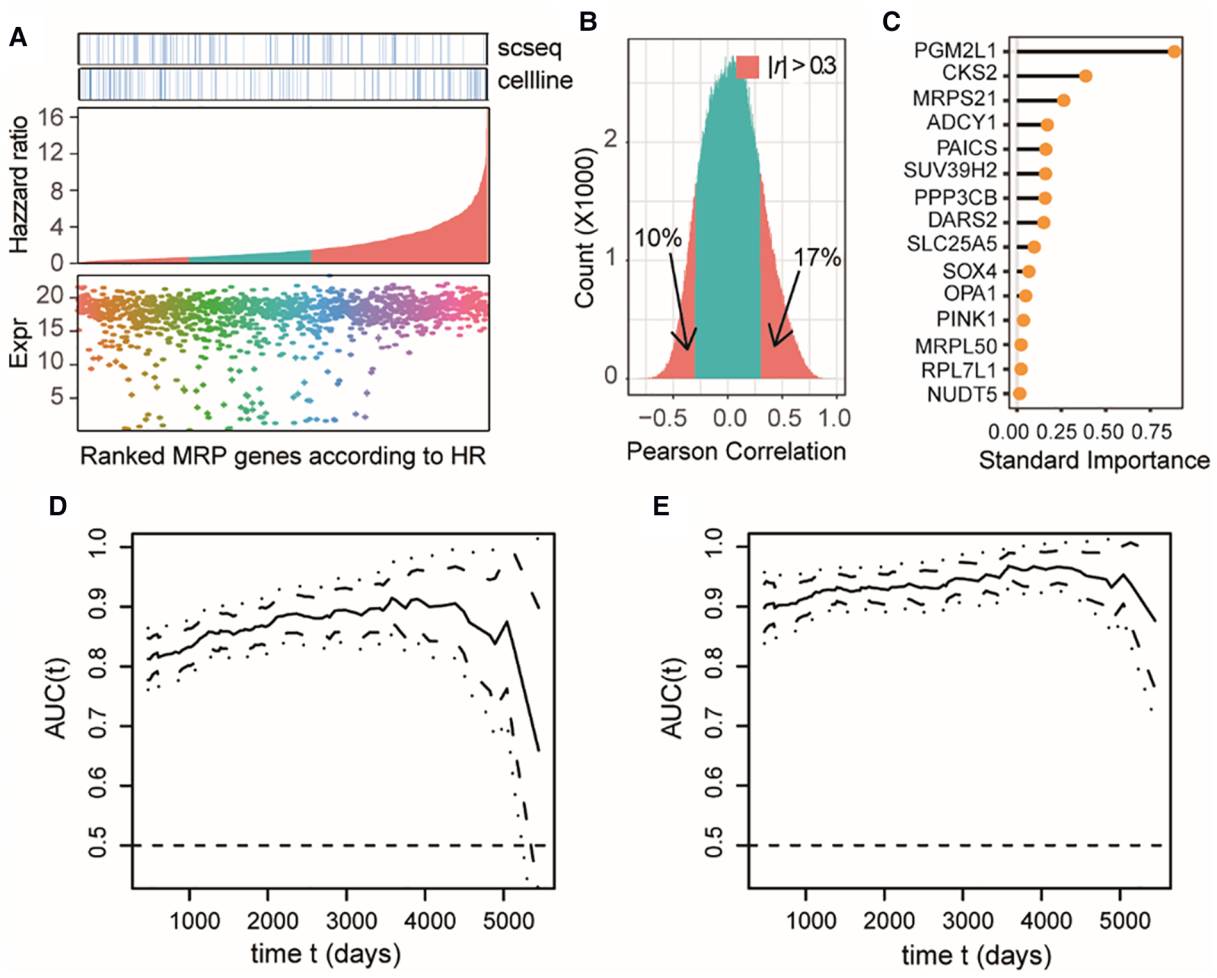
MAP genes can predict clinical outcome of NB patients and exert additive effects. (**A**) Dot plot of expression levels in the bulk tissue dataset of differential MAP genes combining all three datasets (bottom); barplot of the differential MAP genes by ranking the hazzard ratio values obtained by univariant Cox proportional hazard regression analyses (middle); annotation differential MAP genes in the single cell dataset and bulk cell line dataset (top). Red bars in the middle panel indicate significant association with overall survival (*P* < 0.05). (**B**) Histogram of Pearson correlation coefficient between all pairwise of MAP genes by comparing their mRNA level in all samples in the bulk tissue dataset. (**C**) Line plot of ranked standard importance of selected MAP genes by a Lasso regression analysis. (**D,E**) Line plots of the estimated AUC under the time-dependent ROC at each time point with the two-gene model (**D**) and the full-gene model (**E**).

Combining the DEA results from each dataset, we totally identified 1,301 differentially expressed MAP genes associated with NB tumors or clinical subtypes, accounting for approximately 76% of all MAP genes. Most of these differential MAP genes had a high mRNA expression abundance in primary NB tissues (Figure 4A). Univariant Cox proportional hazard regression modeling revealed 880 MAP genes showing significant association with the overall survival (OS) of NB patients (*P *< 0.05). Out of these genes, 542 were identified to be risk factors with higher mRNA levels in NB tumors, with the estimated hazard ratio (HR) ranging from 1.48 to 17.03 (Figure 4A).

We next examined whether these OS-related MAP genes were redundant and to what extent these genes could predict the prognosis of NB. Pearson correlation analysis of the OS-related MAP genes showed that around 27% of all pairwise genes showed a significant correlation in the NB cohort with the absolute values of PCC > 0.3 (Figure 4B), which was a challenge to multivariate regression modeling because of the possible multicollinearity problem. Therefore, we conducted a multivariate regression analysis following an informative feature selection procedure. The feature selection was conducted from 147 differential MAP genes shared by at least two datasets and associated with NB OS. To minimize the adverse impacts raised by overfitting or selection bias, we rated the importance of these genes for clinical outcomes of the patients, dead or censored in our data, using Lasso regression within a framework of 5-fold cross-validation (see Methods). By doing so, we narrowed the long gene list down to 15 (Figure 4C). We constructed a 15-gene model to predict the death event in a time-dependent way with the accuracy ranging from 0.8765 to 0.9678, which was significantly higher than that of the 2-gene model based on the top 2 important genes (*PGM2L1*, *CKS2*) (*t*-test, *P* < 2.2e-16; Figures 4D,E). Moreover, the accuracy of the 2-gene model decreased sharply over time in comparison with the 15-gene model, especially for survival predictions after 4,000 days. We also compared our 15-gene model to those proposed in previous studies: The first one is a three-gene model, including *CKB*, *DST*, and *DUT*; and the second one is a six-gene model, including *CYLD, JAK1, APC, ERH, CNBP*, and *BAX*. In our used NB cohort dataset, we didn’t find the expression of *DST* and *APC*. Therefore, we compared our 15-gene model to the two-gene model (*CKB*, and *DUT*) and the five-gene model (*CYLD*, *JAK1, ERH, CNBP*, and *BAX*). Our 15-gene model perform better than these two models (Supplementary Figures S1, S2). These results together demonstrated that our 15-gene model can accurately predict NB prognosis.

### MAP genes are essential for cancer cells but not for the proliferation of *ex vivo* human primary T cells

**Figure 5:**
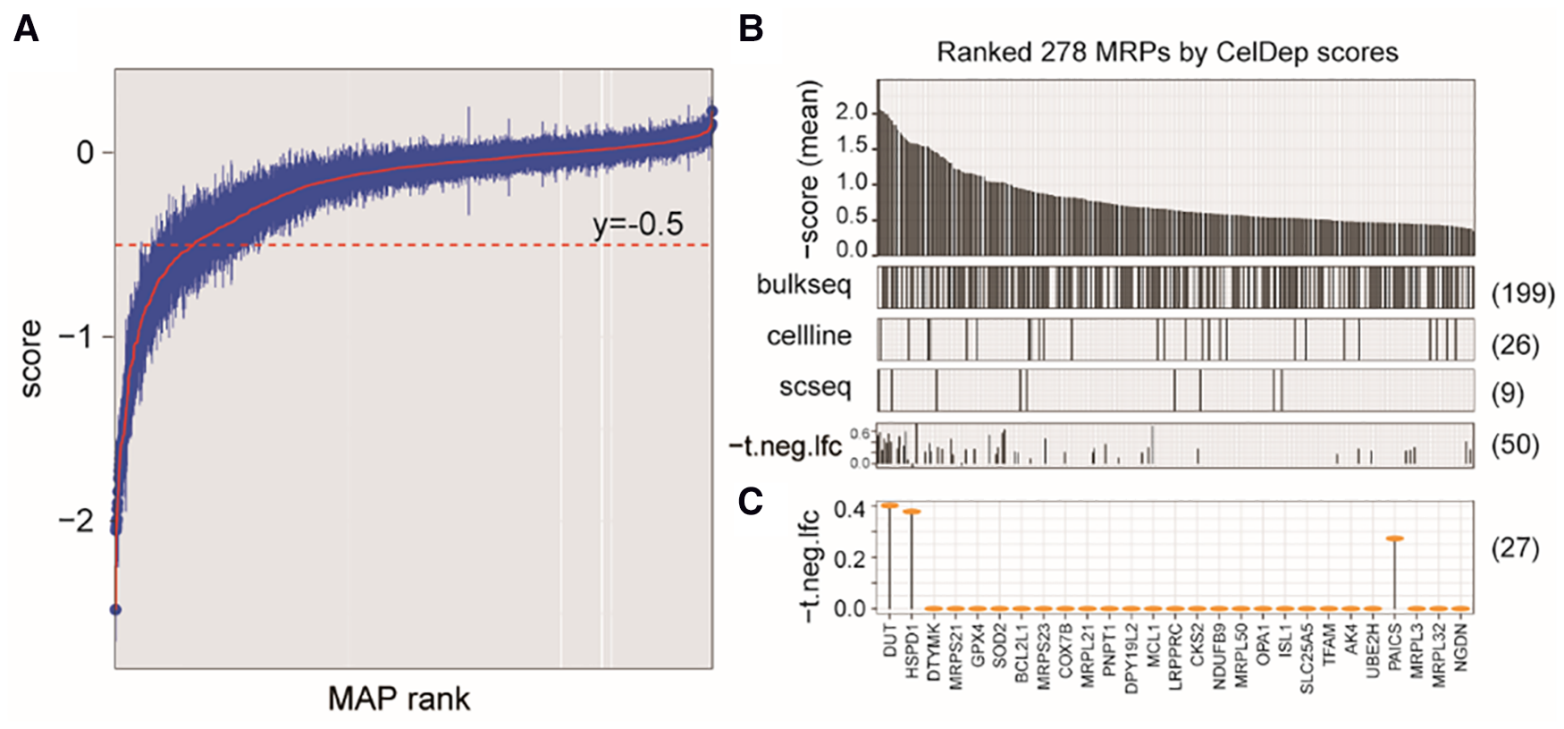
Characterizing MAP genes essential for cancer cells but not for *ex vivo* human primary T cells. (**A**) Simplified boxplots of all MAP genes ranked based on the estimation of gene essentiality in NB cell lines. (**B**) Barplot of ranked MAP genes based on the gene essentiality scores (up panel), indications of overlap with the DEGs identified in three datasets (middle panels), and indications of overlap with the MAPs identified in CRISP-Cas9 dataset derived from T cells (bottom panel)*.* (**C**) Selected MAP gene candidates overlapped between at least two dataset and the T cell dataset.

Because our study revealed that NB cells tend to aberrantly increase mitochondrial synthesis, we next evaluated the potential of targeting MAP genes in NB therapy by analyzing gene dependency assay based on genome-wide CRISPR screening (Dataset 4, see Methods), which is an informative and powerful tool for identifying the gene that are critical for the survival and proliferation of cancer cells. We found a total of 544 MAP genes that were essential for at least one NB cell line, out of which 278 showed higher degree of essentialities that repressed cell survival upon knockout in at least 10 cell lines (Figures 5A,B).

We next evaluated whether targeting these genes in clinical therapeutics, if possible, can disturb the tumor-associated immune microenvironment. We investigated the cellular effects of targeting these genes on the T cells, which play the most important roles in the anti-tumor immune response. Using a genome-wide CRISPR-Cas9 screening data from the primary human T cells (26) (Dataset 5; see Methods), we found that 50 of these MAP genes would not inhibit T cell differentiation *ex vivo* (*P* > 0.05), our of which 27 MAP genes were identified in at least two datasets of Dataset 1–3 used in our study. Of note, our study reproduced *DUT* to be a therapeutic candidate, which was consistent with a previous study showing that targeted inhibition of mitochondrial DNA transcription has shown an anti-tumor effect in mice containing human ovarian carcinoma and colorectal cancer cell xenografts (47). These collective results were suggestive of the potential of targeting MAP genes in NB therapeutics.

### Pan-Cancer analysis of MAP genes

To explore whether some MAPs are always overexpressed or underexpressed in many tumors, we also tested the expression dynamics of these MAP genes in Pan-cancer datasets (see Methods). We performed DEA by comparing the TCGA mRNA expression dataset to the normal tissue expression data from the GTEx. We found that each type of TCGA tumors contained an average of 1,212 differential MAP genes (adjusted *P* < 0.05; Supplementary Figure S3). Of note, approximately 85% of these differential MAP genes were shared in more than 14 cancer types (Supplementary Figure S4). We further found 104 differential MAP genes with high-fold change (>2 fold) and high-frequency (>14 types) in human cancers (Supplementary Figure S5). Interestingly, these MAP genes mainly exhibit two patterns: 43 MAP genes are mainly upregulated in the majority of cancers, and the other genes are mainly downregulated in the majority of cancers. Overlapping these MAP genes with NB-associated differential MAP genes, we found some shared genes including *SOX4, CKS2, SUV39H2, DARS2, PYCR1*, and *PMAIP1*. These results collectively demonstrated the significance of studying MAP genes in human pan-cancers.

## Discussion

A feasible targeted therapy in NB is still urgently needed, especially for patients with high-risk tumors. Because an expanded set of targets would offer additional therapeutic opportunities (26), in-depth and careful reanalysis of publicly available NB-related gene expression profiles at different dimensions will be helpful in identifying potential targets for new treatments. In this study, we conducted a comprehensive and integrated bioinformatics analysis on publicly accessible gene expression data from neuroblastoma (NB) cell lines and primary tissues at both the tissue and single cell level. Our results support the use of MAP genes as potential markers for categorizing and determining the stage of NB tumors, predicting outcomes, and guiding the development of targeted treatments.

With the advance in the development of research techniques in life science, genetic and molecular biomarkers have been frequently examined for risk stratification and prognosis prediction in NB. For instance, the outcome of NB patients has been revealed to be associated with varying molecular signatures, including genetic mutations of specific genes (48), the methylation status of *RB1* and *TDGF1* (49), detectable circulating tumor DNAs in blood biopsy (50), and altered mRNA expression levels of specific gene lists (51–54). In this study, it was found that 542 mitochondria-related protein-encoding genes were associated with the elevated HR of NB patients, which reduced the OS with higher mRNA levels. In addition, this adverse effect on the OS can be addictive.

Targeting mitochondria with activation of the cell death machinery in cancer cells by inhibiting tumor-specific alterations of the mitochondrial metabolism or by stimulating mitochondrial membrane permeabilization has long been thought to be a promising therapeutic approach (55, 56). Targeting mitochondria of cancer cells requires precise delivery of the drugs to the subcellular localizations, which poses challenges for the choice of targets and the design of drug molecules. Our study presented candidates by employing integration of tumor-associated mRNA expression changes at the bulk tissue and single cell resolutions, which extends the choice of molecular targets for targeting mitochondria strategies.

## Data Availability

The datasets presented in this study can be found in online repositories. The names of the repository/repositories and accession number(s) can be found in the article/**Supplementary Material**.
