# Supplementary material for: Mitochondria-associated gene expression perturbation predicts clinical outcomes and shows potential for targeted therapy in neuroblastoma
Source: Front Pediatr. 2023 Mar 21;11:1094926. doi: 10.3389/fped.2023.1094926 (PMC10070980; doi:10.3389/fped.2023.1094926)
Supplement: Supplementary file 5 [file Table5.docx]

Table S1. Differential genes in NB cell lines by comparing to normal cells

Table S2. Differential MAP genes between subgroups in NB cohorts.

Table S3. Differential genes in the single cell RNA-seq dataset of NB tumors.

Figure S1. Line plots of the estimated AUC under the time-dependent ROC at each time point with the two-gene model (DUT, CKB).

Figure S2. Line plots of the estimated AUC under the time-dependent ROC at each time point with the five-gene model (CYLD, JAK1, ERH, CNBP, and BAX).

Figure S3. Bar plot of the number of differential MAP genes in each cancer.

Figure S4. Bar plot of the frequency of MAP genes that were identified to be differentially expressed in TCGA datasets.

Figure S5. Heatmap of differential MAP genes with high-fold change (>2 fold) and high-frequency (>14 cancers) in human cancers.


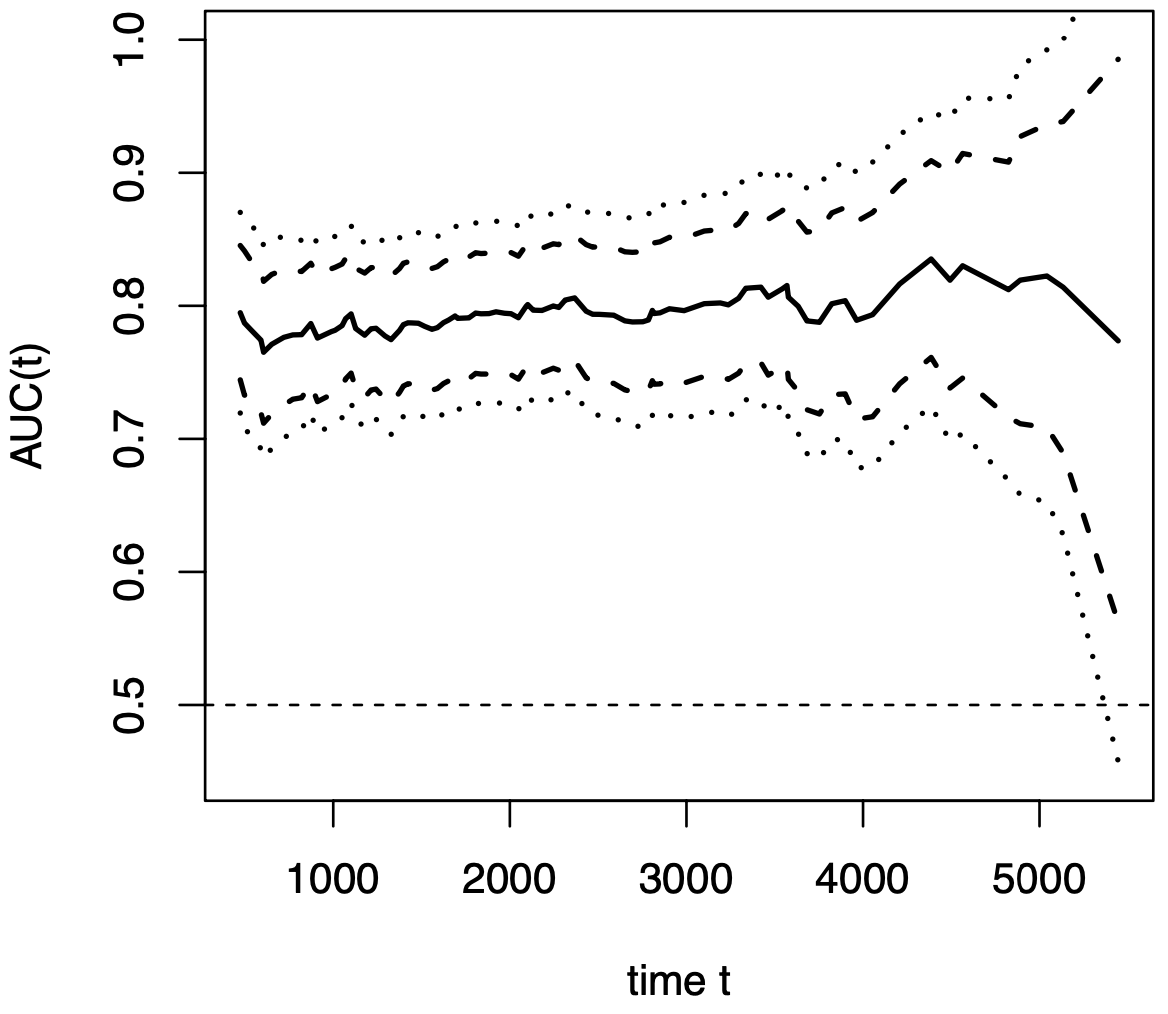


Figure S1. Line plots of the estimated AUC under the time-dependent ROC at each time point with the two-gene model (DUT, CKB).


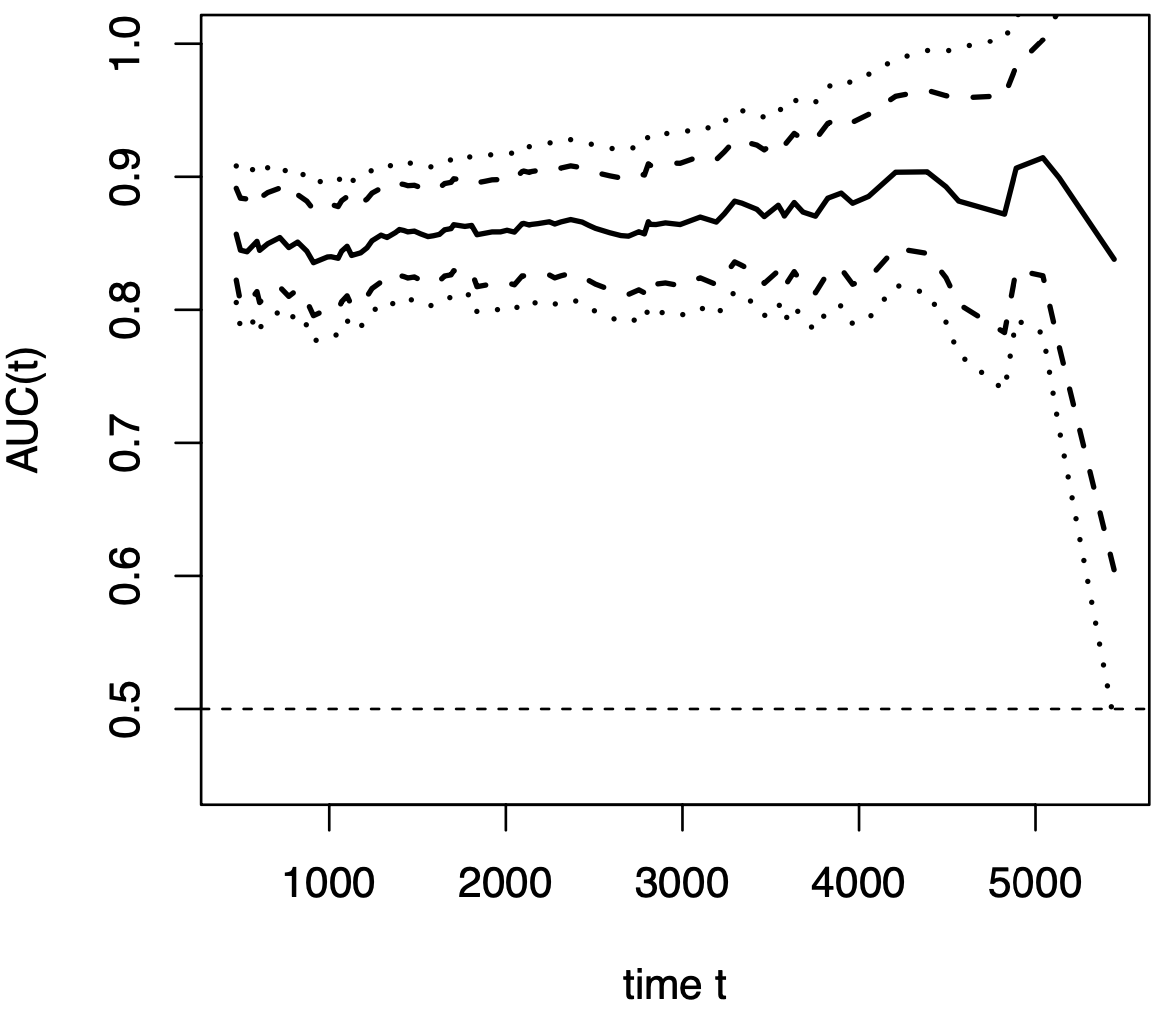


Figure S2. Line plots of the estimated AUC under the time-dependent ROC at each time point with the five-gene model (CYLD, JAK1, ERH, CNBP, and BAX).


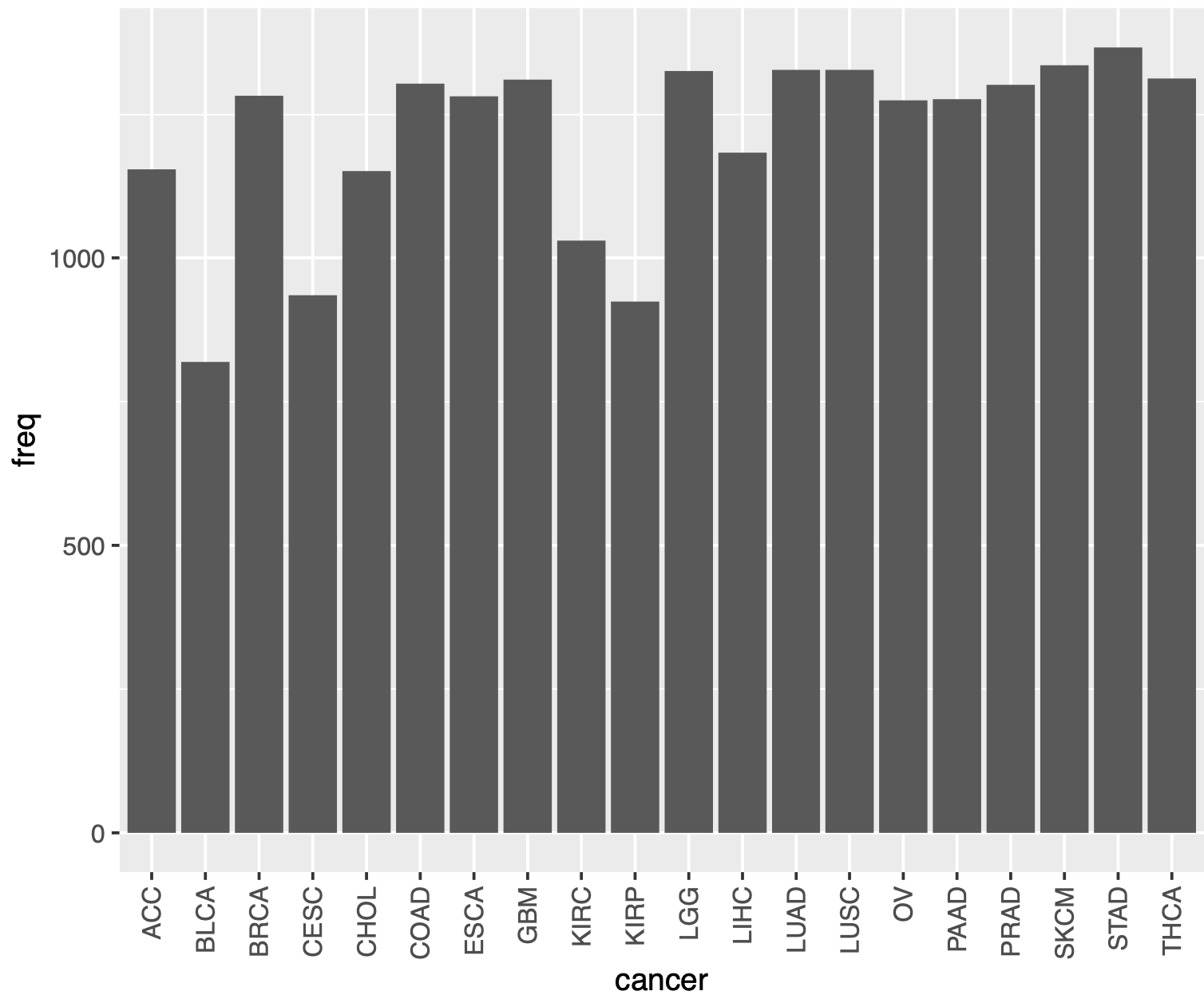


Figure S3. Bar plot of the number of differential MAP genes in each cancer.


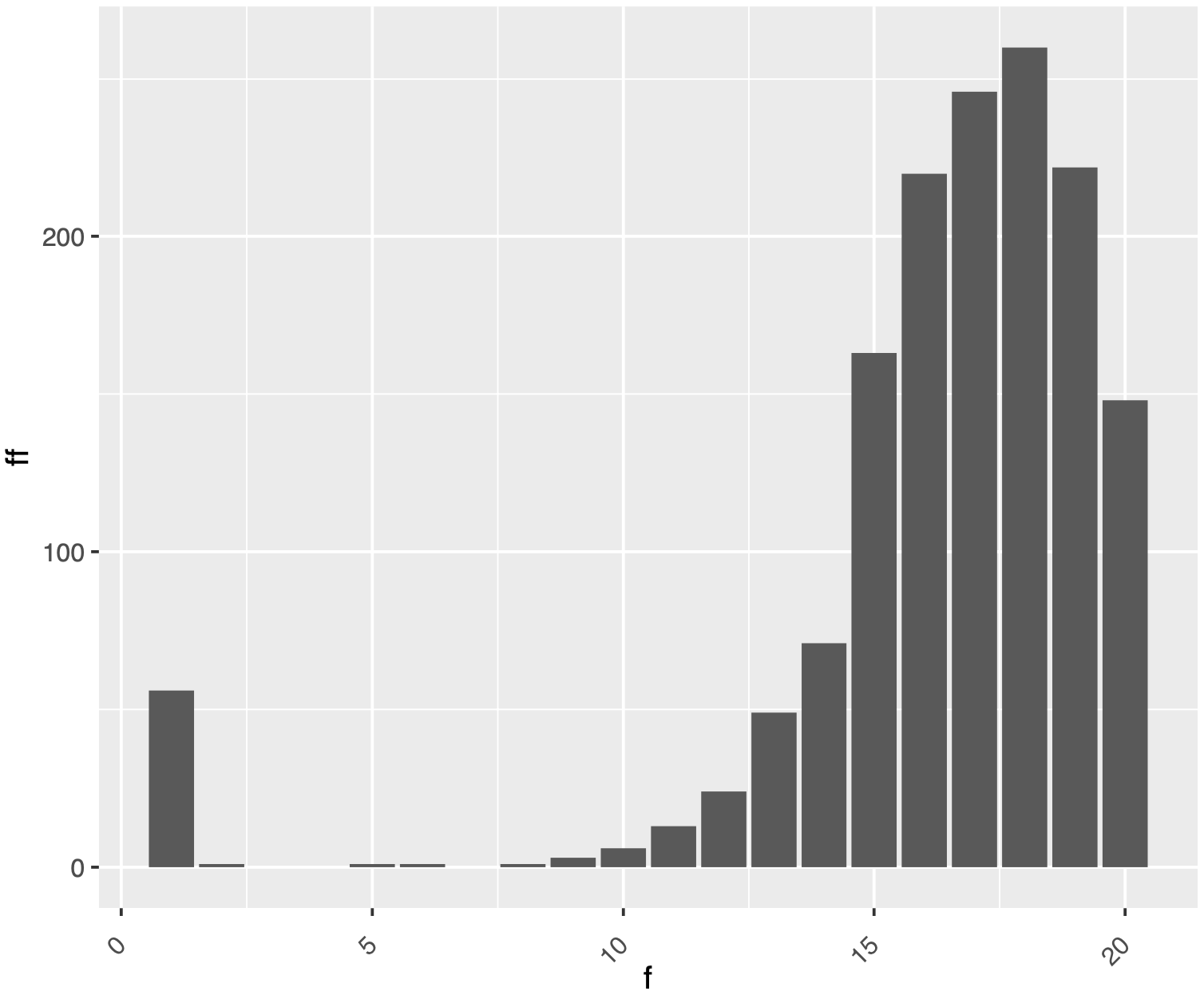


Figure S4. Bar plot of the frequency of MAP genes that were identified to be differentially expressed in TCGA datasets.


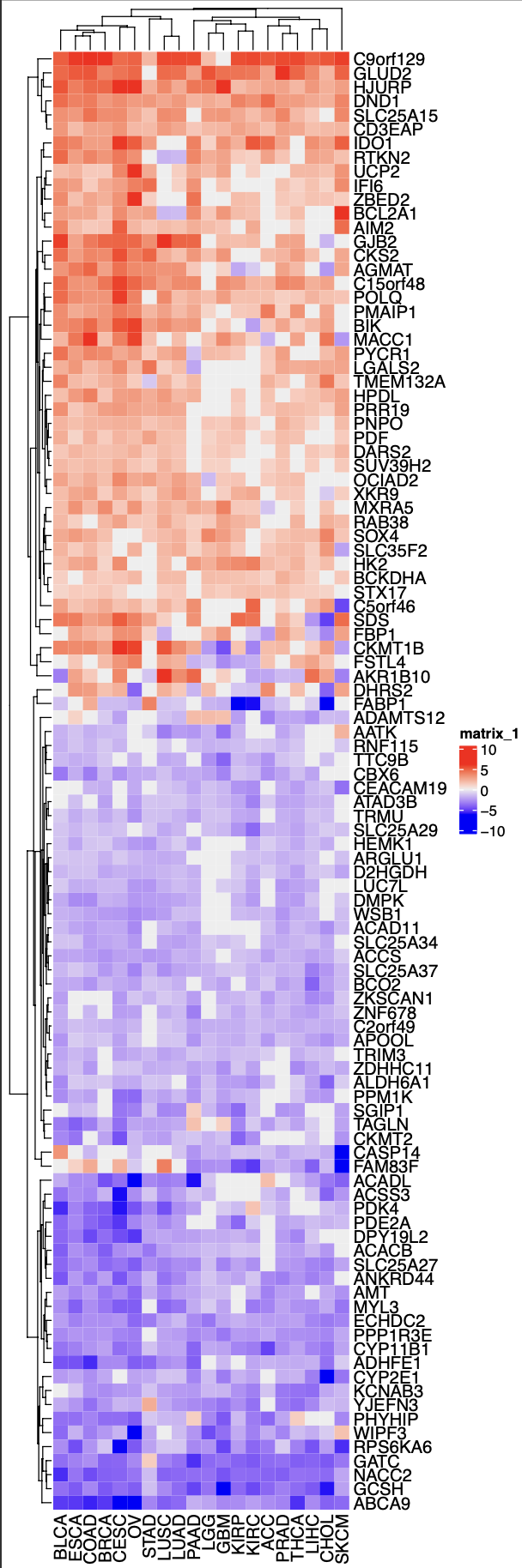


Figure S5. Heatmap of differential MAP genes with high-fold change (>2 fold) and high-frequency (>14 cancers) in human cancers.
